# Review of Sensor Technologies in Animal Breeding: Phenotyping Behaviors of Laying Hens to Select Against Feather Pecking

**DOI:** 10.3390/ani9030108

**Published:** 2019-03-22

**Authors:** Esther D. Ellen, Malou van der Sluis, Janice Siegford, Oleksiy Guzhva, Michael J. Toscano, Jörn Bennewitz, Lisette E. van der Zande, Jerine A. J. van der Eijk, Elske N. de Haas, Tomas Norton, Deborah Piette, Jens Tetens, Britt de Klerk, Bram Visser, T. Bas Rodenburg

**Affiliations:** 1 Animal Breeding and Genomics, Wageningen University & Research, 6700 AH Wageningen, The Netherlands; malou.vandersluis@wur.nl; 2 Department of Animals in Science and Society, Faculty of Veterinary Medicine, Utrecht University, 3508 TD Utrecht, The Netherlands; e.n.dehaas@uu.nl (E.N.d.H.); t.b.rodenburg@uu.nl (T.B.R.); 3 Animal Behavior and Welfare Group, Department of Animal Science, Michigan State University, East Lansing, MI 48824, USA; siegford@msu.edu; 4 Department Biosystems and Technology, Swedish University of Agricultural Sciences, 230 53 Alnarp, Sweden; oleksiy.guzhva@slu.se; 5 Center for Proper Housing: Poultry and Rabbits University of Bern, CH 3052 Zollikofen, Switzerland; michael.toscano@vetsuisse.unibe.ch; 6 Institute of Animal Science, University of Hohenheim, 70599 Stuttgart, Germany; j.bennewitz@uni-hohenheim.de; 7 Adaptation Physiology Group, Wageningen University & Research, 6700 AH Wageningen, The Netherlands; lisette.vanderzande@wur.nl (L.E.v.d.Z.); jerine.vandereijk@wur.nl (J.A.J.v.d.E.); 8 Behavioural Ecology Group, Wageningen University & Research, 6700 AH Wageningen, The Netherlands; 9 Institute for Agricultural and Fisheries Research (ILVO), Animal Sciences Unit, 9090 Melle, Belgium; 10 M3-BIORES, Division Animal and Human Health Engineering, Department of Biosystems, KU Leuven, B-3001 Heverlee, Belgium; tomas.norton@kuleuven.be (T.N.); deborah.piette@kuleuven.be (D.P.); 11 Functional Breeding Group, Department of Animal Sciences, Georg-August University, 37077 Göttingen, Germany; jens.tetens@uni-goettingen.de; 12 Cobb Europe, 5831 GH Boxmeer, The Netherlands; Britt.deKlerk@cobb-europe.com; 13 Hendrix Genetics Research, Technology & Services B.V., 5830 AC Boxmeer, The Netherlands; Bram.Visser@hendrix-genetics.com

**Keywords:** damaging behavior, ultra-wideband, radio frequency identification, computer vision, identification, measuring behavior, -omics, genetic selection

## Abstract

**Simple Summary:**

The European Cooperation in Science and Technology (COST) Action GroupHouseNet aims to provide synergy among scientists to prevent damaging behavior in group-housed pigs and laying hens. One goal of this network is to determine how genetic and genomic tools can be used to breed animals that are less likely to perform damaging behavior on their pen-mates. In this review, the focus is on feather-pecking behavior in laying hens. Reducing feather pecking in large groups of hens is a challenge, because it is difficult to identify and monitor individual birds. However, current developments in sensor technologies and animal breeding have the potential to identify individual animals, monitor individual behavior, and link this information back to the underlying genotype. We describe a combination of sensor technologies and “-omics” approaches that could be used to select against feather-pecking behavior in laying hens.

**Abstract:**

Damaging behaviors, like feather pecking (FP), have large economic and welfare consequences in the commercial laying hen industry. Selective breeding can be used to obtain animals that are less likely to perform damaging behavior on their pen-mates. However, with the growing tendency to keep birds in large groups, identifying specific birds that are performing or receiving FP is difficult. With current developments in sensor technologies, it may now be possible to identify laying hens in large groups that show less FP behavior and select them for breeding. We propose using a combination of sensor technology and genomic methods to identify feather peckers and victims in groups. In this review, we will describe the use of “-omics” approaches to understand FP and give an overview of sensor technologies that can be used for animal monitoring, such as ultra-wideband, radio frequency identification, and computer vision. We will then discuss the identification of indicator traits from both sensor technologies and genomics approaches that can be used to select animals for breeding against damaging behavior.

## 1. Introduction

Damaging behaviors, such as feather pecking (FP) in laying hens, lead to welfare and economic problems in commercial poultry production. FP can be defined as grasping and firmly pulling of feathers of conspecific birds [1]. Savory (1995) categorized five types of bird-to-bird pecking: (1) aggressive pecking, (2) FP without feather removal, (3) feather pulling leading to feather loss, (4) tissue pecking in denuded areas, and (5) vent pecking [1]. More recently, the terms “gentle” and “severe” have been used to differentiate between types of FP. Severe FP, which is essentially Savory’s [1] third category, often leads to mortality and is considered the problematic behavior in terms of damage to the recipient in the laying hen industry. FP occurs worldwide and in all kinds of commercial laying-hen housing systems [2,3]. It is expected that the incidence of damaging behavior like FP will increase, especially due to increased use of housing in large groups as a consequence of bans on conventional cages worldwide and the (expected) ban on beak trimming in many European countries, for example in Sweden, Switzerland, and the Netherlands. Several studies have shown that FP can be reduced using genetic selection [4,5], an approach which offers an auspicious solution to reduce the incidence of FP.

FP is a socially-affected trait, because it depends not only on the hen’s ability to avoid being pecked (direct genetic effect or victim effect; DGE), but also on the pecking behavior of her group mates (indirect genetic effect or actor effect; IGE) [6,7,8]. For plumage condition, it was found that DGEs contribute 6–31% of the total heritable variation, while IGEs contribute 70–94% of total heritable variation [9]. Together they explain 10–54% of total phenotypic variation in a hen’s plumage condition. Therefore, to effectively select against FP behavior, it is important to use a selection method that takes both DGEs and IGEs into account [10].

Associations between FP and other bird behaviors, temperament, or affective states also exist and may be additional targets for genetic selection [11,12]. For example, several studies found that fearful chicks in an open field test showed more FP behavior than adults [12,13]. Furthermore, high locomotor activity and active behavioral responses of birds are related to high levels of FP behavior [14]. Behavioral observations offer a good approximate phenotype for selecting against FP behavior. For instance, the number of FP bouts based on direct observation, has been used to select against FP in laying hens [4]. However, collecting information on activity, fearfulness and FP behavior is time consuming and difficult to apply in commercial breeding situations, because large amounts of data must be collected from many birds. Furthermore, there is a tendency to keep commercial laying hens in large groups (i.e., thousands of birds in one group). Additionally, laying hens are housed at high densities and are difficult to distinguish from one another by human observers or cameras, both of which inherently complicate identifying individual birds for surveillance. Thus, identifying feather peckers and victims and collecting behavioral observations from laying hens housed in commercial systems is challenging. With the use of sensor technologies, such as ultra-wideband (UWB) tracking, computer vision (CV), accelerometers, or radio frequency identification (RFID), there is potential to track and monitor individuals in large groups, and to identify feather peckers and victims [15]. 

A variety of sensor technologies have been developed to monitor activity and behavioral patterns in an automated way. For example, to monitor group activity and presence of birds, sound analysis, image analysis or infrared (IR) thermography are often used [16,17,18]. These technologies are cheap and non-invasive, but they do not allow us to monitor the activity and FP behavior of individual birds. Using CV it may be possible to detect specific behaviors, but it requires the system to distinguish between individual birds and this is often only feasible by marking the birds. This is time-consuming and must be repeated regularly since markings fade over time, and marking a bird may also make her the target of attacks by flock mates. In recent years, accelerometers, RFID, UWB, and geographic information systems (GIS) have been used, fitting birds with a body-worn sensor to monitor individual behavior patterns [19,20,21]. Unfortunately, these systems have not been widely applied in commercial situations, because it is difficult and expensive to upscale to commercial flock size since one sensor per bird is needed. The device often require substantial battery power for transmission of data, and must be able to survive harsh environments during the lifespan of a bird [15,22,23,24]. Additionally, body-worn sensors can have an effect on the well-being of birds, because other hens might be attracted to peck at any visible sensors worn by pen mates [19]. Nevertheless, in a research setting, body-worn sensors can accurately assess behavior and location of hens, with agreement of 95% between automated measurement and labelling by human observers for specific behaviors such as perching, nesting, feeding, drinking, and movement of individual laying hens [23], and 85% accuracy in detecting the location of a hen [25]. To date, no sensor system can accurately and automatically identify and monitor individual hens under commercial situations. However, recent developments in sensor technologies and the combination of different sensor technologies together might offer solutions. New developments in the field of animal breeding can also aid in linking information obtained from sensor technologies to an individual’s genotype. This could help us to identify regions or gene expression patterns that provide further insights into FP behavior and provide tools to reduce the incidence of FP behavior. 

In this review, we will describe the use of “-omics” approaches to understand FP, give an overview of sensor technologies that can be used for phenotyping, discuss the identification of indicator traits from both “-omics” and sensor technologies, and discuss applications to animal breeding.

## 2. Understanding Feather Pecking Through “-Omics” Approaches

The innovative technology platform “-omics” refers to approaches for identifying biomarkers and genes to improve complex traits of interest. The “-omics” approach was first used to define the studies of genomes (genomics) and gene expression (transcriptomics), and is currently also used for studies of proteins (proteomics), lipids (lipidomics), metabolites (metabolomics), and microbiota (microbiomics). So far, most “-omics” studies of FP have focused on genomics.

### 2.1. Genomics Approach

One of the first studies that looked at finding regions in the genome associated with FP in laying hens was done by Buitenhuis et al. [26]. Using a microsatellite-based linkage study in an F2 population established from the cross of commercial lines differing in their propensity for FP, the authors reported three suggestive quantitative trait loci for gentle and one significant quantitative trait locus (QTL) for severe FP [26]. In contrast, another research group identified only a single QTL for FP on a different chromosome in an F2 cross of Red Jungle Fowl and White Leghorn [27]. Several subsequent studies have now been conducted based on single-nucleotide polymorphism (SNP) genotypes. In an across-line association study using a low-density panel of approximately 1000 SNPs, Biscarini et al. (2010) analyzed the DGE as well as the IGE of pen-mates on feather damage [28]. The DGE reflects victim traits, while the IGE reflects feather pecker traits. In total, they identified 81 SNPs associated with IGE for plumage condition in laying hens. A major finding was the implication of a serotonin receptor in expression of FP, supporting earlier evidence for a prominent role of monoamine signaling. However, the number of animals used in this study was limited. 

The most recent studies relating genotype to FP have utilized SNP chips of higher density, providing more detail on the genome. For example, a 60,000 SNP chip (Illumina, 5200 Illumina Way, San Diego, CA, USA) was used in lines divergently selected for high and low FP and an F2 cross of these lines. Mapping results based on selection signatures between the lines [29] and association results from the F2 cross with approximately 900 individuals were jointly analyzed in a meta-study [30], revealing 13 clusters of significantly associated markers and pointing to a candidate gene that might also be related to monoamine signaling. Brinker et al. (2018) performed a genome-wide association study (GWAS) for the DGE and IGE with respect to survival time in crossbred laying hens showing FP [31]. Their results suggest a link with the GABAergic system, which supports existing evidence for the involvement of gamma-aminobutyric acid (GABA; which plays a role in the regulation of neurotransmitters in the brain) in the development of abnormal behaviors [32,33,34]. This is in line with results of gene-set-enrichment and overrepresentation analyses based on the GWAS conducted by Lutz et al. (2017), which also found a link between GABAergic signaling and FP [35]. Further research is needed to investigate the association between the GABAergic system and FP behavior.

### 2.2. Other “-Omics” Approaches

Large-scale transcriptomic studies have so far been performed in chicken lines divergently selected on FP propensity by applying microarray technology. Within the high FP selection line, Labouriau et al. (2009) found significant gene expression differences between extreme feather peckers and birds performing FP at a lower level [36]. These authors proposed the presence of a single allele affecting severe FP. Later, Hughes and Buitenhuis (2010) reported a globally reduced variance of gene expression in high FP birds and found distinct expression patterns associated with gentle and severe FP [37], which supports the hypothesis mentioned above. Brunberg et al. (2011) studied differential hypothalamic gene expression in victims and control birds from FP lines [38] and their findings fit with the commonly proposed hypothesis that FP is a redirected foraging behavior [39,40,41]. Wysocki et al. (2013) identified a number of candidate genes related to neurotransmission and psychopathological disorders including monoamine signaling [42]. The available genomic and transcriptomic studies so far point to a major role of monoamine signaling in FP, which fits well with other available data describing the links between the dopaminergic and serotonergic system and FP [43,44,45,46,47]. Otherwise, however, little congruence has been found among studies, which is to be expected as FP is a complex trait, with heritability ranging from 0.1 to 0.4 [4,48,49,50]. Furthermore, as can be seen from the results of the GWAS experiments conducted so far, a large number of genes are likely contributing to FP phenotypes. Hence, FP is a typical quantitative trait. In order to select against this behavior, breeding values need to be estimated, which requires the continuous recording of FP behavior at the level of the individual. Since FP itself is a difficult to measure trait, the identification of proxy traits is necessary. These can be biomarkers derived from further “-omics” approaches or other indicator traits, such as activity, that can be recorded on a large scale in a breeding enterprise using sensor technologies.

## 3. Sensor Technologies

To date, there are a variety of sensor technologies that have been used to identify and monitor (individual) birds in groups. Overall, there are two main approaches that have been used: body-worn sensor technologies, such as RFID, and remote sensor technologies, such as CV. Below, we will describe the two approaches and the different technologies involved.

### 3.1. Radio Frequency Identification (RFID)

#### 3.1.1. General Introduction to RFID

RFID systems are used for a wide range of applications, including security, logistics, manufacturing, and processing [51]. RFID systems are further used for livestock (reviewed in [52]), particularly in cattle, but also for behavioral research in laying hens. The generic term “RFID” refers to technology that uses radio waves for communication [53,54]. RFID systems generally include tags or transponders, readers (also referred to as interrogators), and a host system [51]. The tags contain an electronic microchip with a unique identification code and an antenna, and can be attached to an animal of interest [53,54]. The reader can retrieve the data from these tags, through communication via antennas working on the same radio frequency, and send this information to a host system [55]. Typically in an RFID system, a record is stored with a time stamp, registered by a certain antenna, and the tag ID [56]. This information is merged with the information on the location of the antenna at which the tag was registered. 

**Figure 1:**
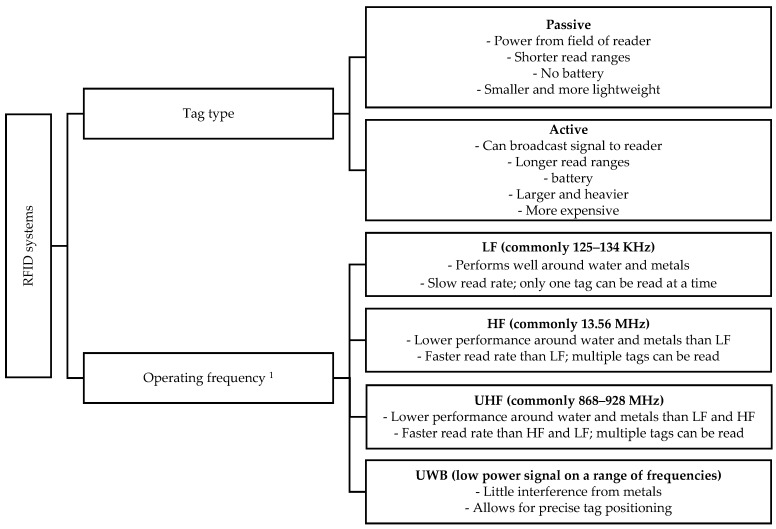
Overview of different radio frequency identification (RFID) systems and their main characteristics (based on [51,52,54,57,58]) ^1^. Microwave frequencies are excluded here, as these are not commonly used for animal tracking. LF: low frequency; HF: high frequency; UHF: ultra-high frequency; UWB: ultra-high frequency.

There are many different types of RFID systems [54], and an important first distinction can be made in the type of tag used. Two main types of tags that will be discussed here are passive tags and active tags (Figure 1). Passive tags obtain power from the field of the reader, as they do not have their own power supply [54]. Active tags like UWB, on the other hand, have a battery that can be used to power the tag circuitry and to actively broadcast a signal to the reader [57]. Generally, active tags have longer read ranges compared to passive tags, but are also heavier and more expensive [51]. Another important distinction among RFID systems can be made based on the operating frequency. Often, four ranges are distinguished: low frequency (LF), high frequency (HF), ultra-high frequency (UHF), and microwave [52]. The four operating frequencies differ in their read ranges and in their sensitivity to the environment. In brief, systems that operate on higher frequencies have longer read ranges and faster communication [52]. However, systems operating on lower frequencies are less sensitive to interference from metals and liquids than higher frequency systems [52].

There are a number of advantages to using RFID for tracking laying hens compared to other methods such as manual observations or video tracking. First, no direct line of sight is required for RFID [54], and the unique identification (ID) code of each tag ensures reliable identification of individuals. Furthermore, multiple tags, or individuals, can be registered within the range of the reader, when higher frequency systems with anti-collision protocols are used [56]. When passive tags are used, the tags can be lightweight, small and long-lasting [51,56]. These tags can be attached on hens, even at young ages. When active tags are required, however, the tags might be too large and heavy to use on young birds. Other constraints on the use of RFID for laying hens include that, depending on the operating frequency, the system can be sensitive to metals and liquids in the environment [52]. This can be problematic in laying hen production systems as they frequently contain a great deal of metal infrastructure in aviary tiers, feed and water lines and manure and egg belts, which must be taken into account when selecting an RFID system. Also, RFID systems can provide information on which individuals are at a specific location, but do not distinguish between different types of behavior being performed in this location. Furthermore, how exact the positional information is depended on whether active or passive systems are used and on the antenna array. Below an overview of applications for laying hens for passive and active systems is given. 

#### 3.1.2. Passive RFID 

Passive RFID has been used in laying hens, for example, to record the use of outdoor ranges [20,59,60,61], general locomotor activity [62] and in lines divergently selected with respect to FP [14], nest box visits [63], and feeding behavior [64]. The performance and suitable applications of RFID systems depend to some extent on the specifications of the systems. Here, some examples of implementations of passive RFID for tracking of laying hen behavior are discussed, for systems operating at different frequencies.

*Low-Frequency (LF) Systems.* Currently, most RFID systems used for laying hens work in the LF range. In a number of studies, passive LF RFID has been used for monitoring pop holes and range use as well as nest use (and see [21] for a review of RFID for recording range and nest box use, e.g., [59,63,65,66]). The general setup in studies of range use is that birds are fitted with an RFID tag and antennas are placed inside the pop hole or on either side of the pop hole. The RFID setup is often validated using video recordings. The reliability and accuracy of the setup differs slightly between studies. For example, Thurner and Wendl (2005) reported an average identification reliability of 97.2% when comparing RFID to video [66], while Gebhardt-Henrich et al. (2014) reported a success rate of registration of 94.3% for exiting the barn and 83.5% for entering the barn [65]. Overall, the RFID setup is noted to be well-suited for monitoring pop hole usage by some authors (e.g., [66]), but in the different studies, some problems were encountered. First, the RFID data required filtering as sometimes false reads occurred, for example when birds entered a pop hole but did not exit on the other side or when birds continuously sat in the pop hole [59]. Second, sometimes birds were not registered when passing the pop hole. This was suggested to be due to multiple hens being present in the pop hole at the same time or due to hens passing the antenna too quickly, at a speed of more than 1.5 meters per second [65,66]. For this reason, Gebhardt-Henrich et al. (2014) suggested reducing the length of the tag’s ID code from 64 to 32 bit, which could increase the maximum speed at which tags would reliably be read when passing the antenna to 3.2 meters per second [65]. Alternatively, this setup could be used for “slower” behaviors such as entries and exits from nests [65]. Indeed, passive LF RFID has also been used successfully to monitor nest occupancy [63,67,68,69]. Other uses of passive LF RFID include monitoring of a hen’s presence in a preference chamber [70] and as identification support for CV approaches [23].

*High-Frequency (HF) Systems.* The data collision problem encountered by LF systems mentioned above when multiple birds are at the same antenna simultaneously may be avoided when higher frequency systems are used. Thurner et al. (2009) and Hartcher et al. (2016) used HF RFID to monitor pop holes use by multiple birds at the same time [61,71]. An identification reliability of between 97.6% and 99.8% was found, depending on the width of the passage [71]. Hartcher et al. (2016) mention no specific success rate for tag registration, but they do conclude that the use of RFID for monitoring multiple individual birds was effective [61]. However, some technical problems (not specified) occurred with the software, and the antennas, power source and computer needed to be in close proximity to each other because of concerns that greater distances (i.e., longer cables) could result in data loss [61]. 

*Ultra-High Frequency (UHF) Systems.* Li et al. (2017) used an UHF RFID system to track feeding and nesting behavior of multiple hens at the same time [64]. In their setup, antennas were placed in feed troughs and nest boxes, and 60 birds were fitted with leg and neck tags. They compared the RFID output to video observations and found an accuracy of the RFID system of 92.1% ± 6.4% (SD) for feeding and 91.4% ± 1.7% (SD) for nesting behavior. The correlation coefficient between RFID and video was 0.98 (slope of 1.01 ± 0.02) for feeding behavior and 0.99 (slope of 0.95 ± 0.01) for nesting behavior. They did find, however, that the detection range was affected by tag position, and the detection range was not uniform over the length of the antenna, with a weaker signal at the cable end of the antenna compared to the free end. Nonetheless, it was concluded that the system was suitable for tracking individual feeding and nesting behaviors of hens. 

#### 3.1.3. Active RFID Systems (Ultra-Wideband)

While the technology provided by HF and UHF systems as described above has been most used for location sensing, UWB, defined as occupying a bandwidth greater than 500 MHz, is an alternative that can improve accuracy in environments where large distances and interference are likely. Operating through signals composed of short duration pulses spread over a wider portion of frequency spectrum [72], UWB is less prone to interference as the wave is not reflected [73], allowing passage through walls and other objects, and does not require a direct line of sight between transmitter and receiver [72]. Hence, UWB technology is considered the most promising type of RFID for indoor use with respect to detecting animal position and tracking movement [72]. Transmission uses relatively little energy [74,75]; hence, UWB is also attractive for long-term applications if the body-worn animal tag is of smaller size and mass. Localization accuracy generally ranges between 12 to 100 cm and is dependent on a variety of factors, including the speed of the animal’s movement, dimensions of interest of the bird’s house, and materials in the tracking area [76,77]. Focusing on dairy cattle in agricultural settings, Porto et al. (2014) evaluated a commercial UWB system and found accuracy within 97 cm for 98% of observations [73]. Similar accuracies were found by others [78]. In another study, a model was developed to measure feeding behavior of dairy cattle using UWB, with 98% accuracy [79].

Focusing on poultry, a recent trial using seven anchors distributed across a 100 m^2^ outside ranging area tracked individual broilers within a semi-commercial mobile housing system and found a mean accuracy of 29 cm [80]. Accuracy was affected by location of the animal within the sensor network (for example, decreased accuracy at the edges of the field), rain, and presence of vegetation [80]. Focusing on the interior environment, Rodenburg et al. (2017) used an UWB system to track individual hens within different test situations [25]. Comparison between UWB and an automated video system found the technologies yielded similar results, although the UWB system was better at tracking multiple birds simultaneously compared to video tracking. To date, UWB systems have not been used to track hens in commercial systems, although the low energy usage, the ability to be less affected by interference, and promising results in other environments suggest the technology has potential in these conditions. To date, the relatively high cost of the system and the sensitivity to environmental conditions, however, might make the system more suitable for research and development environments, rather than for commercial farms.

#### 3.1.4. Application of RFID to FP

There are several requirements that RFID systems must meet for effective monitoring of FP in group-housed laying hens. First, individual recognition of hens is required to assign phenotypes to individual birds. Given that the tags that are used in RFID systems each have their own identification code [53,54], identification of individual birds is ensured. Furthermore, because FP is a social behavior, FP can only be studied in groups. Although passive LF RFID systems generally do not allow for reading multiple tags at one antenna at the same time, passive higher frequency and UWB systems can register multiple tags (Figure 1; [80]). Therefore, when passive higher frequency or UWB systems are used, multiple individuals can be monitored and tracked while they are housed in groups. However, for passive RFID systems, there is a tradeoff between fast read rates and performance around metal and water (Figure 1; [58]). When laying hens are studied in commercial environments that include metal, interference of the RFID signals can result, especially at higher frequencies. To avoid interference by the environment, UWB systems can be used, as these are less sensitive to interference from metals [58]. However, a major downside to the use of RFID for monitoring FP is that RFID cannot directly recognize FP behavior. RFID systems can register the location of animals, but cannot indicate what behavior the animals show in this location. Therefore, to assess FP with RFID, either proxies for FP or combinations with other sensor technologies are required (see Section 4 and Section 5). One trait that has potential as a proxy for FP in laying hens is locomotor activity, as Kjaer (2009) showed that birds from a high FP line are more active than birds from a low FP line [14]. Passive RFID can be used to monitor activity of individual laying hens by positioning antennas throughout the area available to the birds. The detail of the positional information can then be adjusted by changing the number and read range of the antennas, to provide the desired degree of detail with which the use of space, and consequently activity, of the hens can be determined. UWB can also be used for activity monitoring, by determining the distances moved by hens based on the longitudinal information on location. In this way, RFID systems could potentially be used to assess FP in laying hens.

### 3.2. Computer Vision

#### 3.2.1. General Introduction to The Computer Vision Approach and Its Key-Aspects

The core definition of CV continues to change with the development of the research field and practical applications of this technology in different areas of livestock production. This includes the general object recognition, behavioral studies, health or welfare assessments, spatial distribution, and activity indices. 

As Ballard and Brown (1982) wrote, “CV is the construction of explicit, meaningful descriptions of physical objects from images” [81]. However, continuous monitoring of animal health and welfare requires a slightly different definition, as it includes the need to make a decision about the animal’s state based on recorded/analyzed information. In this situation, the definition of CV from Shapiro and Stockman (2001) is a more appropriate one: “CV is about making useful decisions about real physical objects and scenes based on images” [82]. In both cases, images used in CV could include visible light, IR, or 3D images or video (Table 1).

Considering the multifactorial nature of FP, CV systems capable of tracking, identifying and monitoring the behavior of individual birds in large groups are not yet fully developed [83]. From a technical perspective, CV applications for tracking multiple individuals in real time is a daunting task for several reasons. One of the main problems is initial object recognition-seperating birds (through segmentation) from a uniform background in a production environment, where litter could have the same pixel values as feathers, is computationally challenging. Another important aspect of a functional CV system is the ability to handle the visual occlusion that occurs among animals at high stocking density, for example when birds pile on each other, obscuring all or part of other birds. Further, birds tend to flock together while maintaining relatively low velocity in space, such as when they forage or preen. However, when individuals do move, they will display features that can be used to define different activities such as rapid spatial movements and directional changes, though these will also complicate the initial scene evaluation necessary for image or video pre-processing such as optical flow or changes in spatiotemporal constancy.

Most CV applications for animal monitoring rely heavily on a set of core-components required for successful completion of the desired tasks: image acquisition, object recognition and tracking, and, ultimately, visual data analysis. Table 1 provides an overview of the main types of cameras used for image acquisition as well as possible shortcomings under different research scenarios, such as type of building environment, research question/hypothesis, number of individuals, and desired features. Enviornmental factors like dust and ammonia affect the longevity of cameras. However environment-proof housing (casing) of the cameras can overcome these problems. Another challenge is the packaging of the images and the size of them after packaging. Using CV, large file sizes are the norm, therefore, a good storage and data infrastructure plan are needed. The use of “real-time” algorithms can solve this: analyzing images and only storing the relevant information. The images can then be deleted straight away. This, however, requires computational power on site. 

The choice of methodology for object detection is task-specific. However, modern CV applications quite often combine several techniques to achieve better accuracy and performance. The use of convolutional neural networks (CNNs) and their variations is a widely applied approach, especially if the task requires real or near real-time implementation. The four main technical approaches used for object detection are:

1. Scene-based: works better in controlled environments with uniform illumination and scene parameters that could be easily altered;
2. Shape-based: utilizes geometrical characteristics of the object-to-find for example lines, points, edges and is usually applied as the number of filters scanning the image in a sliding-window manner. Heavily relies on image acquisition method, image quality, and number of objects to detect;
3. Motion-based: uses the temporal difference between corresponding frames to find changes in pixel velocity, creating “shadow maps” with object’s positions or activity indices; mostly used for area supervision;
4. Appearance-based: utilizes such image properties as the number of channels, color intensity, hue, to detect the desired object. Heavily relies on image acquisition method and number of objects to detect.

#### 3.2.2. Overview of Recent Developments in CV Related to Monitoring Larger Groups of Individuals

Aydin (2017) proposed a 3D camera-based solution for assessing inactivity in broilers to detect lameness [84]. The core idea was to measure broilers’ posture and activity indices in a test corridor, where a Kinect V1 (Microsoft^®^) camera was mounted, providing a top-down overview over the passing broilers (*n* = 250). The number of lying events (NOL) and the latency to lie down (LTL) in the observed broilers were correctly classified by the proposed 3D vision monitoring system and showed high positive correlations with gait scores (R^2^ = 0.93 and 0.95, respectively). The system has potential to be a crucial stepping stone in the development of applications suitable for real-time monitoring of complex health and behavior related issues in birds/laying hens/poultry. However, in order for it to be viable for large-scale use, several issues should be addressed:

- The Field of View (FOV) of the Kinect V1 camera is relatively small (for example, 1.5 × 1.5 m at 2-m height). For large rooms and areal scanning, the camera would need to be moved around at a pre-defined speed to capture the full area of interest, which could affect both scene reconstruction from depth data as well as object detection. An alternative would be to have several cameras with overlapping FOV. However, linking the cameras together is still a challenge;
- Computational costs (due to image size and algorithm complexity) and limits to cable extension (as the camera requires a stable bandwidth for data flow) could be a problem if used on farm;
- The features defining welfare/health-related issues should be consistent across variable environments (e.g., different lightning conditions, levels of dust) and be of a type that allow fast processing in scenes with high stocking density.

Nakarmi et al. (2014) proposed another potentially interesting solution in their automated tracking and behavior quantification system for laying hens, combining RFID and CV [23]. The setup included an experimental area 1.2 m × 1.2 m in size, equipped with 20 antennas (installed under the floor surface) for the identification of individual birds and a 3D-camera (Microsoft^®^ Kinect V1) for image data acquisition. The system proved capable of tracking/identifying up to ten individuals in this limited space with an average 95% accuracy when compared to manual observations. An algorithm was used for object detection in frame-to-frame correspondence, while an RFID-transponder placed on a lower part of a hen’s leg provided individual identification. This sensor-fusion approach, where different sensors provide different pieces of information necessary for continuous assessment of the production environment, could minimise the potential drawbacks of using only one type of technology. However, for such a system to work in large groups of individuals, advanced logistics are needed for hardware and software infrastructure which might result in additional economic investments.

Fortunately, recent developments related to optimising hardware architecture allow more computationally-heavy tasks to be performed at lower costs. This allows for integration of both supervised and unsupervised machine learning algorithms in many monitoring tasks and provides tools needed for large-scale scene assessments and analysis of complex biological data. Wang et al. (2016) proposed a hen-tracking algorithm based on a hybrid support vector machine (HSVM) [85]. The tracking algorithm used consisted of three steps: initialization, tracking and updating of an object. The proposed system performed with an average accuracy of 75% compared to other supervised machine learning systems (e.g., particle filter algorithm, mean shift algorithm or standard support vector machine (SVM) with single or double arguments). Performance of the HSVM, however, was heavily affected by the physical scale of the observed images, number of objects in the scene, and directional changes of moving hens. Large-scale applications will require a mixed segmentation/analysis approach capable of working with objects of different shape and size, as well as robust filtration for overcoming the physical and CV-specific occlusion occurring in scenes where high flocking densities result in densely packed or overlapping birds.

Zhuang et al. (2018) used a different approach to create an early warning algorithm for detecting sick broilers that utilised unsupervised k-means clustering for object detection [86]. K-means clustering partitions a data space into *k* clusters, each cluster with a mean value. Each individual in the cluster is placed in the cluster closest to the cluster’s mean value [87]. For further analysis of health-related issues, the skeleton attitude angle and back concavity were used as defining features. In later stages of analysis, SVM was used for classification of parameters linked to health and welfare. Another interesting approach based on 3D cameras that is being used for weight prediction of broilers at both individual and group level was developed by Mortensen et al. (2016). Their system showed high correlations between objects’ detected area and weight, allowing consistent, continuous monitoring of growth rate and indirect feed consumption rates [88].

Dawkins et al. (2013) used optical flow methodology for welfare assessment of larger groups of hens. However, analyzing 15-minute long sessions per hour might not be sufficient for continuous monitoring of flock activity [89]. Previously used optical flow approaches also do not provide the level of detail and individual tracking capability necessary for quantitative behavioral studies, since it operates at a general scene level. Thus, while cost-efficient for large area observations, optical flow lacks the flexibility of somewhat more complicated CV methods. This method does, however, have potential for flock and group level assessment of general activity and distribution. If groups are housed according to genetic line or family, this information can still be useful for animal breeding. 

Zaninelli et al. [24,90,91] made several attempts to develop and improve a monitoring system for laying hens based on IR technology and image pattern recognition. Their approaches required a top-mounted thermal camera and were capable of processing acquired images in real-time while the proposed algorithm detected objects within an image based on gradient changes in pixel temperature values. The total area of the experimental room in their studies was 8 m^2^, while the FOV of the camera was 110 cm × 150 cm. The so-called recording/detection window was set at two seconds. During this time, the system would take a series of images and detect objects based on threshold deviations from mean floor temperature. The authors suggested that the camera could be mounted in a portable way in order to move between different areas of the house to reduce the need for and cost of extra cameras. This approach could be considered a first step in finding a cost-effective, computationally cheap system for monitoring distribution of groups of birds. However, to make such system viable, one would need to ensure faster recording intervals for detection of rapid movements of individuals as well as a combination with more dynamic CV approaches for real-time monitoring of changes in group dynamics. One could also consider adding identification of individuals using passive RFID-tags. Another consideration specific to using IR/thermal camera-based systems is that threshold values will be highly dependent on a large number of environmental and management factors (e.g., temperature at the room level, humidity, dust particles, breed of bird, color of feathers, feeding regime, health status; see Table 1). Therefore, using IR/thermal camera-based systems will require extensive calibration.

## 4. Use of Sensor Technologies in Different Applications

Despite the aforementioned rapid advances in hardware and analytical processing, surveillance efforts continue to be limited by physical characteristics of laying hens and the manner in which they are housed, especially the relatively high stocking densities. These limitations offer a partial explanation for why progression with precision livestock farming in poultry has been considerably slower than in other livestock species such as dairy cattle and swine. Furthermore, hens are also frequently housed in tiered systems made largely of metal, which can interfere with signal transmission using RFID. Differentiating individuals is also limited by nearly uniform color patterns across the flock, particularly in white lines. Additionally, the relatively small size of laying hens (typically less than 2 kg) severely limits the mass of the equipment that can be mounted on animals (i.e., 100 g or less assuming a 2 kg hen and a 5% body mass guideline [21]), and thus consequently requires unique characteristics for battery power and miniaturization. 

Besides problems with visualizing animals, the physical arrangement of laying hen housing, particularly of non-cage systems, further complicates monitoring. Unlike dairy cattle and swine, laying hens routinely move in all three dimensions, traveling through vertical space as they navigate up and down tiers or perches in their environment. Hens normally roost at night on elevated structures with provision of perches being legally mandated in the European Union, though they spend most of their waking hours at ground level performing species-specific behaviors such as dust bathing or ground scratching and ground pecking. Aviary systems, a type of non-cage system that consists of several stacked tiers reaching up to 3.5 m high, with feed and water distributed throughout, seek to take advantage of how hens use both horizontal and vertical dimensions. Recent data suggest that hens are highly consistent in how they use such multi-dimensional space [92] which is in turn affected by physical injury such as keel fractures [93]. Regarding FP, there is evidence that most of the FP takes place in the litter area, so increased use of the litter area could also be used as a proxy measure for FP [14]. Thus it is important for monitoring systems to possess the capacity to observe hens in three dimensions. 

Outside of housing constraints, similar to other species, laying hens have destructive tendencies and investigate by pecking, pulling apart whatever they can. While hens lack the strength of larger livestock, their sharp claws and beak afford a unique capacity to destroy equipment that must be considered while developing equipment to be worn by hens or stationary equipment that is accessible to hens in the barn. 

**Figure 2:**
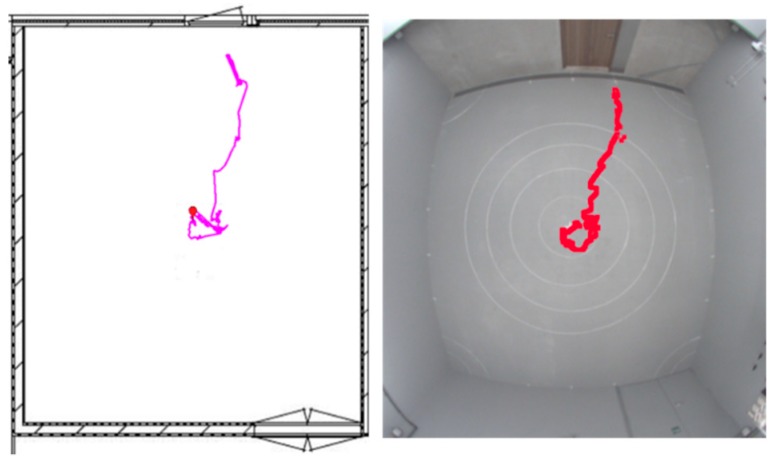
Tracking an individual laying hen using ultra-wideband (UWB) tracking (left panel) and video tracking (right panel).

Given the challenges highlighted above, different settings impose different constraints on what monitoring systems can be used and the type of information that can be collected. Methods for tracking individual birds using RFID or UWB tracking systems are currently being developed. One such example, PhenoLab, has been used for automatic tracking of laying hens in floor pens. In the PhenoLab project, UWB tracking using TrackLab (Noldus Information Technology, Wageningen, the Netherlands) was compared with video tracking of individual hens decoded using Ethovision from the same company [25]. Here, hens were outfitted with an active UWB tag in a small backpack and the location of each bird was detected using stationary sensing beacons that combined time and angle of arrival of the signal. As can be seen from Figure 2, both systems yielded very similar results and the UWB system was able to detect the location of the bird with an 85% accuracy compared to the human observer [25]. 

Furthermore, PhenoLab has been used to explore behavioral differences between lines of hens selected for high and low FP. These hens originated from the lines used by Kjaer [14]. The primary goal was to determine if previously found differences in activity between the lines could be detected automatically, with the high FP line being hyperactive [14]. This was indeed the case, with hens of the high FP line moving almost twice the distance as hens of the low FP line or the unselected control line. Furthermore, based on the tracking data, individual differences within lines could also be investigated with birds characterized as feather peckers (based on video observations) showing more activity than birds characterized as victims [25]. This underlines that activity level could be used as a proxy measure for FP, with high activity linked to an increased risk of being a feather pecker.

A second example of tracking individual poultry comes from a free range broiler study, where ranging behavior of broilers was studied using an UWB tracking system [80,94]. The system was successful in registering the position of the tag in 68% of the cases during validation and had an accuracy of 0.29 m. This meant that the actual location was on average within 0.29 m from the measured location. It performed well across varied environmental conditions and thus offers a good opportunity for outdoor tracking of hens, including examining proximity of hens in relation to each other.

Alternatively, when focusing on tracking hens in a commercial house where birds are able to move in three dimensional space, trading knowledge of specific spatial coordinates for identification of hen location within specific zones or areas is an option that can be used to overcome limitations in resolution. Rufener et al. (2018) used specifically placed emitters of IR beams encoded with one of five zone-specific codes that were recognized by receivers worn by the hens [92]. Data were limited to recognizing the hens’ presence within one of these five broad areas, but the resulting information demonstrated highly consistent movement and location patterns of individual hens from which useful metrics could be generated [92]. Furthermore, movement and location patterns of hens appeared to change in response to age and physical injury [93]. However, a major drawback of the IR system is that tracking reliability is dependent on the quality and quantity of coverage provided by the emitters. For the particular housing system used by Rufener et al. (submitted), hens’ entry into and thus presence within a particular zone were limited by the arrangement of the tiers, which were stacked directly on top of one another, a common but not universal arrangement for tiered aviaries [93]. Hence, for this system, placement of emitters could be concentrated at points of access to each zone and the litter area, which was open. Nonetheless, even when less coverage is needed, assuming each emitter could provide coverage over a 1.0 m × 2.3 m space, such a system requires substantial cabling and installation time, resulting in limited utility. Easier installation and effectiveness across more types of systems, including aviaries with an offset arrangement, is needed to make IR systems more universally useful. Ongoing trials using UWB signals generated by cables laid within the barn appear to generate similar recognition of hen movement among zone-specific areas. Preliminary trials have yielded results comparable to those generated by Rufener et al. [92]. As the strength of the generating signal can be adjusted, additional cables deployed at key resources, for example along the feeder or drinking lines, can likely be used to aid in the identification of specific behaviors important to welfare or production efficiency. 

With these types of systems, it will increasingly become possible to identify specific behaviors of animals and make assessments in terms of how hens respond to specific events. For instance, using RFID in combination with load cells on perches, it is possible to describe frequency, duration, and circadian patterns of perch use by individual laying hens [95], though, with such a system it is not currently possible to determine whether these hens are sitting, standing, resting, or sleeping soundly while on the perches. In other cases, RFID antenna at key resources such as food and water can assess proximity of birds to these resources [96]; however, without additional measurements, it is not possible to determine if the birds, and which birds, are actually using the resources they are near and how much food or water they might be ingesting. Perhaps a combination could be made with sound analysis of pecking sounds, as proposed by Aydin and Berckmans [97]. Alternatively RFID/UWB could be combined with body worn sensors to gain more detailed behavioral information. It is possible to use the intensities and patterns of activity collected from three-axis accelerometers to train software to detect specific behaviors of laying hens such as dust bathing or feeding [98] or falls or collisions within a tiered aviary system [99]. It should also be possible to train such systems to detect injurious behaviors such as pecks directed toward other birds either aggressively or related to FP due to characteristic quick motions and positioning of the head and body when delivering these pecks. These can be distinguished from slower, less forceful pecks down toward feed or up toward water nipples.

Ultimately, as illustrated by several of the preceding examples, the use of body-worn sensors in poultry may be the most powerful on a commercial scale when coupled with other technologies. For example, a combination of RFID/UWB and accelerometers have the most potential to monitor FP in large groups of poultry. This is because RFID/UWB could give the location of a bird, whereas accelerometers will indicate the directional movement and speed, which can indicate FP. Realistically, sensors will likely need to be deployed on a sentinel population of birds within the larger flocks, though research is needed to ensure adequate numbers of animals and an effective selection protocol.

**Figure 3:**
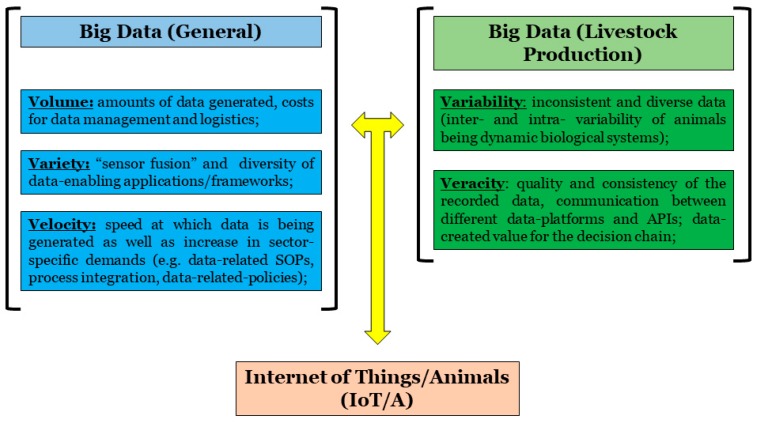
Big data in livestock production.

For the successful and efficient development of new sensors, improvement and validation of existing ones or communicative integration between different application programming interfaces, clear visualization of the data chain is required. Most sensor-based systems for monitoring animal health, performance and welfare generate tremendous amounts of data. However, for farmers and breeding companies to turn data into something of value for decision making, a highly functioning data-driven infrastructure connecting all on-farm devices and management systems is required [100]. According to Verdouw et al. (2015) three main steps need to be implemented in any data-related framework/management system for it to be viable for use on commercial farms or breeding facilities [101]. The main steps include (1) sensing and continuous monitoring, (2) analytics and support for decision making, and (3) standard operating procedures. Therefore, Internet of things (IoT) and big data (BD) are swiftly becoming essential areas in poultry production, including the 5V’s and the soon-to-be Internet of animals (IoA) (Figure 3).

## 5. Identification of Indicator Traits from Sensor and “-Omics” Technologies

We are now at a point where both sensor technology and ‘-omics’ approaches have the potential to provide a large amount of data at the level of the individual hen, which can be used to understand and selectively breed against FP. For example, the high and low FP lines [62], selected based on whether they show high or low FP behavior, have now been characterized in genomic and transcriptomic studies. These studies have added to our knowledge of the mechanisms underlying FP behavior. The phenotypes recorded in these studies were mainly based on direct observation of the behavior. For breeding purposes, however, easy-to-measure indicator (proxy) traits are needed, which can arise from both “-omics” and sensor technology approaches. Using sensor technology, we can now record detailed information on hens from the various FP lines, creating an individual behavioral profile that describes a hen’s activity, location and proximity to other individuals. If we use both sensor and “-omics” technological approaches in a breeding population, we can link the genomic data to the ethological data, and define the genomic profile of individuals that show the desired behavior (for example, low or no FP). As a prerequisite, however, we need to determine genetic parameters for each indicator trait derived from sensor data, especially the genetic correlations between indicator and target traits. Once thought impossible, this approach may now be feasible, because breeding companies have begun to routinely genotype their breeding stock, and they are also investing in methods for automatic phenotyping. Once the desired genomic profile has been defined, we can test whether selecting for this profile will reduce FP by breeding a next generation based on genomic selection and then phenotyping it with the same tools used to phenotype the parent stock. Thus, we feel that a combined sensor and “-omics” approach has great promise to select against complex behavioral traits that involve multiple individual animals in a group, such as FP in laying hens. More specifically for monitoring FP in large groups of birds, a combination of CV, RFID/UWB, and probably accelerometers has the most potential.

## 6. Conclusions

Reducing FP behavior is important in commercial poultry production. The current developments in “-omics” and sensor technologies offer possible solutions to reduce FP. We argue that a combined sensor and “-omics” approach has great promise to contribute to the breeding program to select against complex behavioral traits such as FP, particularly when sensor types, such as CV and RFID/UWB, are combined.

## Figures and Tables

**Table 1:** Image acquisition layout and an overview of the different challenges using different types of cameras.

Type of Camera	Camera Subtype	Factors to Consider during the Recording	Factors Potentially Affecting Data Quality
**Infrared (IR)**	**Operating range (affects the sensitivity of measurement)** Near-IR; Short wave (SWIR); Medium wave (MWIR); Long wave (LWIR);	Environmental factors (e.g., temperature, humidity, dust particles), angle and distance of measurement, reflective properties of the measured object (e.g., dry or wet feathers)	
**2D**	**Network/IP cameras** Cheap, used for surveillance, often robust and have protected casing, compress images, great range of in-built functions, embedded hardware is capable of some processing, remote access for multiple users;	**Sensor type** CMOS: Less expensive, high dynamic range (HDR), no blooming, making it perfect for varying objects and scenes with varying illumination, low power consumption; CCD: More expensive, can capture more light, lower noise factor, higher fill and color reliability, making it perfect for less dynamic tasks and low-light conditions; **Type of image** Monochrome: Higher sensitivity and detail level, more difficult to process and analyze if the scene illumination varies; Color: RGB channels, possible to enhance based on pixel values, good for scenes with varying conditions; **Frames Rate (FPS) capacity** High Frame Rate = more images captured per second = faster sensor = higher data volume; Trade-off between desired complexity of behavior/parameter and image size; **Resolution** Resolution = (Object Size/Detail size)²	Camera calibration and scene reconstruction Scene Illumination Number of animals and background clutter Lens properties Occlusion Scale Object “deformation”
	**Industrial/Computer vision (CV) cameras** Area Scan: Allows in-depth scene inspection as the image is recorded and processed “at once”; Line Scan: High-speed tasks, quality control, data are captured line-by-line and then reconstructed into the whole image; Expensive, images recorded in “raw” format and transferred to PC for processing, complex infrastructure for setup;		
**3D**	**Stereo cameras** Cheap, two or more lenses, depth range for recording depends on the distance between lenses, limited extensibility;		
	**Continuous Wave Time of Flight (ToF) cameras** Relatively cheap, emits continuous wave modulated light which returns back with depth data, usually lower sensor resolution, a wide range of functions (e.g., motion capture, scene reconstruction, object scanning);		
	**Structured Light Cameras** Cheapest, use an active stereovision approach (the known IR-pattern is projected onto the object of interest, and the depth data is calculated based on distortion occurring on the collision of IR-pattern and objects’ shape)		

IR: Infrared; IP: internet protocol; CV: computer vision; CMOS: complementary metal-oxide-semiconductor; CCD: charge-coupled devices; FPS: frames per second.
